# Combined pharmacological activation of AMPK and PPAR *δ* potentiates the effects of exercise in trained mice

**DOI:** 10.14814/phy2.12625

**Published:** 2016-03-20

**Authors:** Mark Christian C. Manio, Kazuo Inoue, Mina Fujitani, Shigenobu Matsumura, Tohru Fushiki

**Affiliations:** ^1^ Graduate School of AgricultureDivision of Food Science and BiotechnologyLaboratory of Nutrition ChemistryKyoto UniversityKyotoJapan

**Keywords:** AMPK and PPAR delta, endurance exercise, gene expression, metabolism, substrate shift

## Abstract

The combined activation of the cellular energy sensor AMP‐activated protein kinase (AMPK) and the nuclear transcription factor peroxisome proliferator‐activated receptor delta (PPAR *δ*) has been demonstrated to improve endurance and muscle function by mimicking the effects of exercise training. However, their combined pharmacological activation with exercise training has not been explored. Balb/c mice were trained on a treadmill and administered both the AMPK activator AICAR and the PPAR *δ* agonist GW0742 for 4 weeks. AICAR treatment potentiated endurance, but the combination of AICAR and GW0742 further potentiated endurance and increased all running parameters significantly relative to exercised and nonexercised groups (138–179% and 355% increase in running time, respectively). Despite the lack of change in basal whole‐body metabolism, a significant shift to fat as the main energy source with a decline in carbohydrate utilization was observed upon indirect calorimetry analysis at the period near exhaustion. Increased energy substrates before exercise, and elevated muscle nonesterified fatty acids (NEFA) and elevated muscle glycogen at exhaustion were observed together with increased PDK4 mRNA expression. Citrate synthase activity was elevated in AICAR‐treated groups, while PGC‐1*α* protein level tended to be increased in GW0742‐treated groups. At exhaustion, *Pgc1a* was robustly upregulated together with *Pdk4*,* Cd36*, and *Lpl* in the muscle. A robust upregulation of *Pgc1a* and a downregulation in *Chrebp* were observed in the liver. Our data show that combined pharmacological activation of AMPK and PPAR *δ* potentiates endurance in trained mice by transcriptional changes in muscle and liver, increased available energy substrates, delayed hypoglycemia through glycogen sparing accompanied by increased NEFA availability, and improved substrate shift from carbohydrate to fat.

## Introduction

AMPK is a cellular energy sensor activated during conditions of stress via upstream kinases. It is sensitive to the ratio of AMP to ATP during periods of energy deprivation such as in exercise, hypoxia, and starvation (Shackelford and Shaw, 2009). Reactive oxygen species produced in the mitochondria during hypoxia and influx of calcium during muscle contraction or nerve stimulation in other organs also activate AMPK (Hawley et al. 2005; Jensen et al. 2007; Mungai et al. 2011). The phosphorylation of AMPK at its *α* subunit leads to a cascade of events involving direct immediate control of metabolism by promoting energy production while inhibiting energy expensive anabolic processes (Winder and Hardie 1996; Kurth‐Kraczek et al. 1999; Horman et al. 2002). It also functions indirectly in energy homeostasis by regulating gene expression through activation or repression of transcription (Jäger et al. 2007; Yang et al. 2009; Chen et al. 2010; Li et al. 2011). Its beneficial role has been widely recognized because of its central role in metabolism particularly in improving fat oxidation and glucose uptake in the muscle, and improvement of lipid handling and oxidation in both fat depots and liver, thereby ameliorating metabolic disorders.

PPARs are nuclear transcription factors which function as lipid sensors leading to transcriptional programming within cells. The PPAR isotypes are present in most tissues but vary in abundance and function as defined by their target genes. PPAR*γ* is abundantly expressed in the adipose tissues orchestrating adipogenic differentiation, lipogenesis, and insulin sensitivity (Desvergne and Wahli 1999; Ahmadian et al. 2013). PPAR*α* is highly expressed in the liver and other oxidative tissues such as the heart and skeletal muscle where it regulates oxidative metabolism of fat (Desvergne and Wahli 1999; Lefebvre et al. 2006). PPAR*δ*, highly abundant in the skeletal muscle and present in other metabolic organs albeit in moderate expression, has been shown to regulate the change to an oxidative phenotype characterized by increased fatty acid oxidation, preferential use of fatty acids as substrate, but with improved glucose uptake (Krämer et al. 2007; Reilly and Lee 2008). The potential of PPAR*δ* in the management of metabolic disorders has been recognized by many researchers owing to its positive role in both fat and glucose utilization. Moreover, its role in the improvement of exercise performance has been demonstrated both by genetic manipulation and by pharmacological activation (Wang et al. 2004; Narkar et al. 2008; Gan et al. 2011).

The interaction of AMPK and PPAR*δ* has been investigated in different contexts. For example, despite the lack of exercise training, mice that underwent 4 weeks of treatment with AICAR together with PPAR*δ* selective agonist GW501516 had increased expression of genes related to endurance training thus termed “exercise mimetics” (Narkar et al. 2008). In another study, the mouse model of muscle dystrophy *Mdx* mouse showed improved muscle functional performance with exercise and the abovementioned drugs in some tests albeit not improving in the running test (Bueno Júnior et al. 2012). It has been demonstrated that AMPK and PPAR*δ*, but not PPAR*α*, physically interact leading to an increase in glucose oxidation via the upregulation of the lactate dehydrogenase B (LDHB) gene associated with improved exercise performance (Narkar et al. 2008; Gan et al. 2011). Much work is still needed to reveal the complexity of AMPK and PPAR*δ* interaction as well as interaction with other PPAR isotypes.

The combined pharmacological activation of AMPK and PPAR*δ* raised a question whether their activators could further improve endurance above that brought about by an exercise training regimen in healthy individuals. In connection to this, considering the consistent demand for ergogenic aids and recovery supplements (Maughan 1999), the use of these chemicals as doping substances being reported was not surprising despite insufficient safety and efficacy studies in humans. To be able to identify food components and natural compounds with similar benefits, we initially determined the effects of combined AMPK and PPAR*δ* activation in trained mice. We show that considerable improvements in endurance could be attained with combined pharmacological activation in healthy exercise‐trained mice.

## Materials and Methods

### Animals and drugs

Male 7‐week‐old Balb/c mice (Shimizu Laboratory Supplies Co. Ltd., Kyoto, Japan) were utilized in the study. The animals were divided into five groups and acclimatized to the housing environment 7 days before the experimental treatments while receiving daily handling and i.p. injection of saline (5 mL kg BW^−1^) to eliminate the effect of stress at the start of the treatments. All animals were housed in a room maintained at 22 ± 0.5°C, 50% humidity, and a light–dark cycle of 12 h (6:00 lights on; 18:00 lights off). Mice had free access to standard diet for mature rodents (D10012m, AIN‐93M; Research Diets, New Brunswick, NJ) and water. Mice in one group received the vehicle DMSO (Wako Pure Chemicals, Osaka, Japan) at 2.5% concentration in physiological saline and were not exercise‐trained (hereby termed SED). The other four groups received treatment together with exercise training. The vehicle group (hereby termed V) received the vehicle. The PPAR*δ* group (hereby termed G) received the potent and selective agonist GW0742 (5 mg kg BW^−1^, s.c.; Sta. Cruz Biotechnology, Santa Cruz, CA). The AMPK group (hereby termed A) received the activator AICAR (500 mg kg BW^−1^, i.p.; Wako Pure Chemicals), and the combined pharmacological activation group (hereby termed A+G) received both GW0742 and AICAR at the same dosage as groups G and A. The administered dose of AICAR was similar to Narkar et al. (2008). GW0742 dosage used was similar to the dosage of another PPAR*δ* selective and potent agonist GW501516 used in the same paper. All administered solutions were prepared to provide a target dosage in a volume of 5 mL kg BW^−1^. Injections and/or exercise training were conducted daily between 8:00 and 12:00 for 4 weeks. Mice assigned to sedentary indirect calorimetry were placed in metabolic chambers from day 26 to acclimatize as described in the succeeding section. Mice assigned to exercise‐to‐exhaustion test with indirect calorimetry were left undisturbed for 3 days. Animal experiments were in accordance to the Kyoto University Guidelines for the Ethical Treatment of Laboratory Animals.

### Exercise training protocol

**Figure 1:**
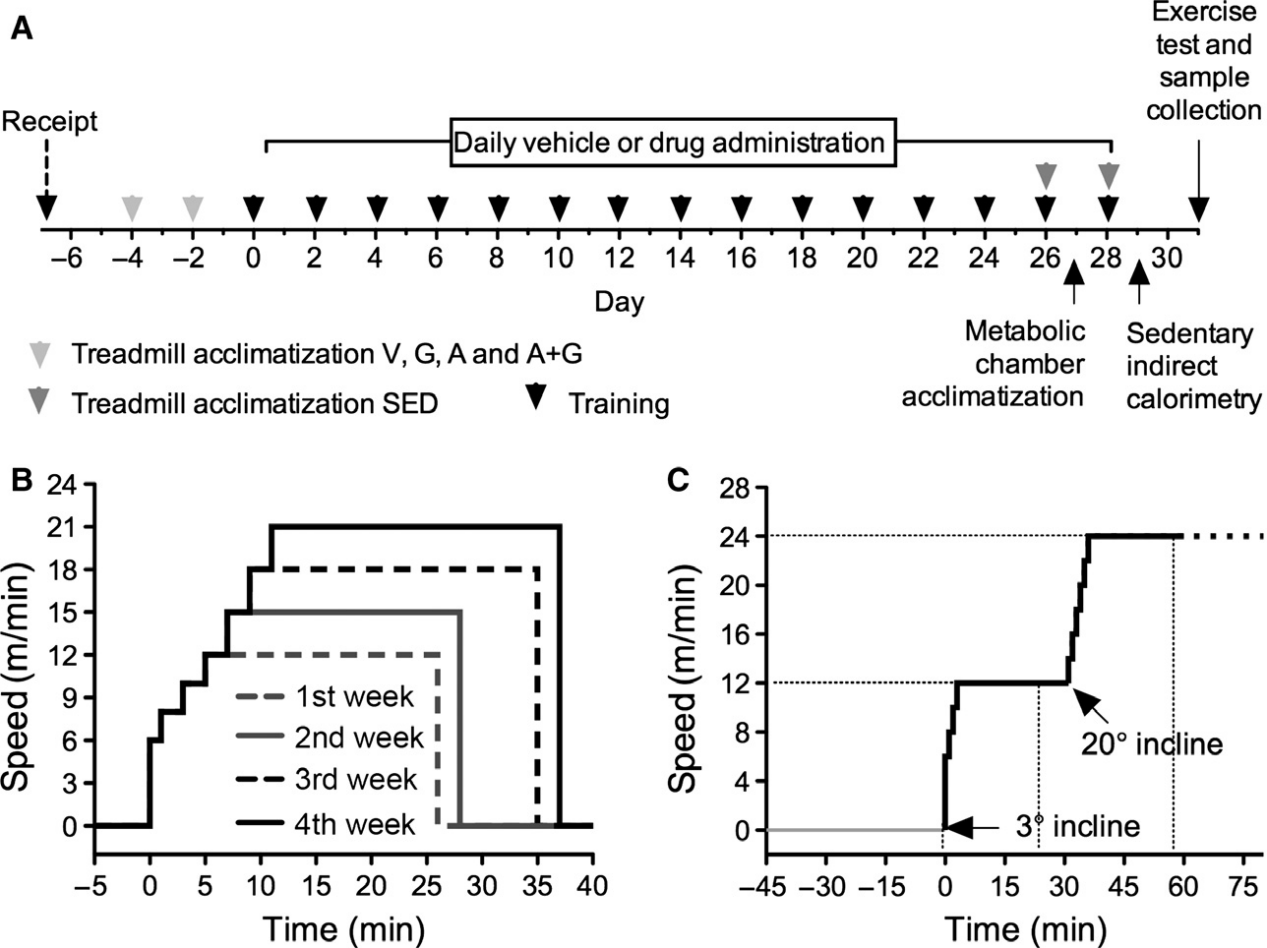
Schedule and exercise protocols. (A) Experiment schedule, (B) training protocol on the treadmill, (C) exercise‐to‐exhaustion test with indirect calorimetry protocol.

The experiment schedule is depicted in Figure 1A. In brief, mice in the exercise‐trained groups (V, G, A and A+G) were accustomed to a rodent treadmill (MK‐680; Muromachi, Tokyo, Japan) by walking for 15 min on 2 alternate days before commencing with the actual training program. Training was conducted on alternate days for 4 weeks with the last two training sets done on a rodent treadmill enclosed in a metabolic chamber (Columbus Instruments, Columbus, OH) coupled to a mass spectrometer (ARCO‐2000; Arco System, Tokyo, Japan). A weekly increasing intensity protocol was followed (Fig. 1B). Briefly, warm‐up was conducted at the following program: 6 m min^−1^ for 1 min, 8 m min^−1^ for 2 min followed by 10 m min^−1^ for 2 min. Following warm‐up, the intensity was set to 12 m min^−1^ for 20 min. On the second week, the warm‐up program included 12 m min^−1^ for 2 min and followed by the intensity of 15 m min^−1^ for 20 min. On the third week, the warm‐up program included 15 m min^−1^ for 2 min and followed by the intensity of 18 m min^−1^ for 25 min. Finally, on the fourth week, the warm‐up program included 18 m min^−1^ for 2 min and followed by the intensity of 21 m min^−1^ for 25 min. An electric stimulus of 0.5 V was employed to force the mice to run. All mice in the exercise‐trained groups were able to fully comply with the training protocol. Mice in the SED group were acclimatized on the rodent treadmill only on the last two training days (Fig. 1A).

### Sedentary indirect calorimetry

Mice were placed in metabolic chambers on day 26 to acclimatize as well as eliminate the effect of environmental stress (Fig. 1A). After the final exercise training bout and drug treatment, sedentary indirect calorimetry were conducted on mice designated to the before‐exercise group. In brief, mice were kept in metabolic chambers and indirect calorimetry was conducted on day 29 for 48 h. Respiratory gases (O_2_ and CO_2_) from each chamber were measured and respiratory quotient (RQ), oxygen consumption, carbohydrate oxidation, and fat oxidation were calculated relative to the body weight of mice. The room was maintained at 25 ± 2°C at a relative humidity of 40–90%. The same diet and water were supplied ad libitum during measurement. The apparatus had 16 lanes each connected to a metabolic chamber (internal dimension: 10 × 16 × 8 cm). Mass spectrometer (ARCO‐2000; Arco System), and air‐lane switching system sampler (ARCO‐2000‐GS‐16; Arco System) were used to measure oxygen consumption and RQ. An activity meter on top of each chamber counted movement. Fat oxidation and carbohydrate oxidation was automatically calculated from the amount of oxygen consumed and respiratory gas exchange ratio using the ARCO‐2000 software coupled to the equipment according to the formula by Frayn (1983), while energy expenditure was calculated using the formula by Lusk (1924). Data during the last 24 h were analyzed and presented.

Two hours prior to sacrifice, food was removed from each chamber. Mice were sacrificed by decapitation and blood was collected. Blood was centrifuged and the collected serum was stored at −80°C for subsequent analyses. Gastrocnemius and a piece of the liver were clamp‐frozen in liquid nitrogen and stored at −80°C for metabolite quantification. Data from these samples were referred in‐text as before exercise or pre‐exercise test data. Weights of right gastrocnemius, right epididymal fat, and whole liver were recorded.

### Exercise‐to‐exhaustion test with indirect calorimetry

Three days after the final exercise training bout and drug treatment, exercise‐to‐exhaustion test was conducted (Fig. 1C). In brief, mice were weighed and placed in an airtight indirect calorimetry treadmill chamber undisturbed for 1.5 h. The treadmill was set at an inclination of 3° and running was commenced at an initial intensity of 6 m min^−1^ gradually increasing by 1 m min^−1^ every 30 sec until the intensity of 12 m min^−1^ was reached totaling to 3 min. This intensity was maintained for 27 min. The inclination was increased to 20° followed by increasing the intensity by 2 m min^−1^ every min until the intensity of 24 m min^−1^ was reached. An electric stimulus of 0.5 V was employed to force the mice to run. This was kept constant until the mice were deemed unable to continue running. Exhaustion was defined as remaining on the shocker plate for 10 sec in spite of momentary increases in electrical stimulus together with tapping on the chamber walls as an auditory stimulus. Indirect calorimetry was conducted before and during exercise using the same system in the sedentary‐state indirect calorimetry section.

At the point of exhaustion, mice were immediately sacrificed and samples were collected similar to mice in the sedentary state. In addition, quadriceps and a piece of the liver were stored in RNAlater RNA Stabilization Reagent (Qiagen, Hilden, Germany) according to the manufacturer's instructions until total RNA extraction. Data from these samples were referred in‐text as after exhaustion or postexercise test data.

### Serum glucose, triglycerides, and nonesterified fatty acids

Serum glucose concentration was measured using an enzymatic colorimetric test kit (Glucose C I Test Wako; Wako Pure Chemicals Industries, Osaka, Japan) on a 96‐multiwell plate reader. Serum triglycerides (TG) and nonesterified fatty acids (NEFA) were measured using the Triglyceride E and NEFA C Test Kits, respectively (Wako Pure Chemicals Industries).

### Muscle and liver glycogen

Frozen gastrocnemii and liver samples were powdered in liquid nitrogen‐submerged mortar and pestle. Glycogen was extracted using the following method in our laboratory. In brief, weighed samples approximately 100 mg was digested in 0.3 mL of 30% KOH solution in a heating block set at 100°C for 30 min. The resulting digest was cooled on ice followed by the addition of 50 *μ*L saturated Na_2_SO_4_ and 0.5 mL ice‐cold ethanol. The mixture was vortexed and spun down at 2300 *g* for 5 min. The glycogen precipitate was dissolved in 200 *μ*L distilled water followed by 250 *μ*L ice‐cold ethanol precipitation. The mixture was centrifuged to pellet the glycogen and the supernatant discarded. To convert the glycogen to glucose units, 0.6 mL of 0.6 mol L^−1^ HCl was added and heated on a heating block set at 100°C for 2 h. Measurement of glucose was similar to that of serum glucose. Glucose was converted to glycogen by factoring the water eliminated if glucose units were polymerized.

### Muscle and liver nonesterified fatty acids

From the powdered tissue samples, approximately 50 mg were weighed. One mL of Folch reagent (chloroform:methanol, 2:1) was added to the sample and then vortexed. The mixture was incubated at 4°C for 16 h. To obtain the lipid containing fraction, 0.2 mL of 4 mmol L^−1^ MgCl_2_ was added then vortexed. The mixture was centrifuged at 1200 *g* for 1 h at 4°C. From the lower chloroform layer containing the extracted lipids, 0.2 mL was collected and the solvent evaporated. The desolvated lipids were resuspended in 0.1 mL 10% triton‐X in isopropanol. NEFA was measured using the NEFA C Test kit.

### Reverse transcriptase quantitative polymerase chain reaction (RT‐qPCR)

Pre‐exercise clamp‐frozen gastrocnemius samples and postexercise quadriceps and liver stored in RNAlater were powdered in liquid nitrogen. Total RNA was extracted using a combination of TriPure Isolation Reagent (Roche, Mannheim, Germany) and RNeasy Mini Kit (Qiagen). In brief, approximately 100 mg of the sample was mixed with 1 mL TriPure reagent followed by brief sonication. The mixture was clarified by centrifugation and 0.2 mL chloroform was added to the supernatant. The mixture was vortexed followed by centrifugation at 12,000 *g* for 15 min at 4°C. Equal parts of 70% ethanol and supernatant were combined, mixed, and then transferred to an RNeasy spin column. The succeeding steps were according to the manufacturer's instructions with DNase I (Qiagen) digestion. Total RNA was reverse transcribed with M‐MLV reverse transcriptase (Promega, Madison, WI) and RNase Inhibitor (Toyobo, Osaka, Japan). Messenger RNA expression levels were measured from signals from Universal ProbeLibrary probes (Roche).

Intron spanning oligonucleotide primer sets of mouse genes were designed using the program at the website of Roche Universal ProbeLibrary Assay Design Center and the predicted amplicons were validated using the program at the BLAST website of the National Institutes of Health. Primer sequences are as follows: hypoxanthine–guanine phosphoribosyltransferase (HPRT) as an internal control (Fwd: 5′‐TCCTCCTCAGACCGCTTTT‐3′; Rev: 5′‐CCTGGTTCATCATCGCTAATC‐3′), peroxisome proliferator‐activated receptor 1‐alpha transcript variant 1 (PCG1a/PPARGC1a) (Fwd: 5′‐TGTGGAACTCTCTGGAACTGC‐3′; Rev: 5′‐AGGGTTATCTTGGTTGGCTTTA‐3′), pyruvate dehydrogenase kinase 4 (PDK4) (Fwd: 5′‐CGCTTAGTGAACACTCCTTCG‐3′; Rev: 5′‐CTTCTGGGCTCTTCTCATGG‐3′), lipoprotein lipase (LPL) (Fwd: 5′‐GCTCATGATGAAGCTTAAGTGGA‐3′; Rev: 5′‐TCCCTAGCACAGAAGATGACC‐3′), fatty acid translocase (FAT/CD36) (Fwd: 5′‐TTGTACCTATACTGTGGCTAAATGAGA ‐3′; Rev: 5′‐CTTGTGTTTTGAACATTTCTGCTT‐3′), muscle carnitine palmitoyltransferase 1 (CPT1b) (Fwd: 5′‐GCCCATGTGCTCCTACCA‐3′; Rev: 5′‐CTCTGAGAGGTGCTGTAGCAAG‐3′), uncoupling protein 3 (UCP3) (Fwd: 5′‐TGCTGGAGTCTCACCTGTTTAC‐3′; Rev: 5′‐CGGGTCTTTACCACATCCAC‐3′), vascular endothelial growth factor A (VEGFa) (Fwd: 5′‐ACTGGACCCTGGCTTTACTG‐3′; Rev: 5′‐TCTGCTCTCCTTCTGTCGTG‐3′), hormone sensitive lipase (LIPE/HSL) (Fwd: 5′‐AGCGCTGGAGGAGTGTTTT‐3′; Rev: 5′‐CCGCTCTCCAGTTGAACC‐3′), citrate synthase (CS) (Fwd: 5′‐CTGCCTGAGGGCTTATTTTG‐3′; Rev: 5′‐CATTCTCGTGAGAGCCAAGAC‐3′), PPAR*δ* (PPARD) (Fwd: 5′‐GAAGTGGCCATGGGTGAC‐3′; Rev: 5′‐GAGGAAGGGGAGGAATTCTG‐3′), glucose transporter type 4 (GLUT4) (Fwd: 5′‐GACGGACACTCCATCTGTTG‐3′; Rev: 5′‐GCCACGATGGAGACATAGC‐3′), adipose triglyceride lipase (ATGL) (Fwd: 5′‐CGGGTA GCATCTGCCAGTA‐3′; Rev: 5′‐CAGTTCCACCTGCTCAGACA‐3′), 3‐oxoacid CoA‐transferase (OXCT1) (Fwd: 5′‐AGGCCTGAC TGTTGATGACA‐3′; Rev: 5′‐CTGCATTGGCATGAGGTTT‐3′), muscle glycogen phosphorylase (PGYM) (Fwd: 5′‐AGTGGAGGACGTGGAAAGG‐3′; Rev: 5′‐GCTCAGGAATTCGGTCGTAG‐3′), muscle glycogen synthase (GSY1) (Fwd: 5′‐GGGGTCTTCCCCTCCTACTA‐3′; Rev: 5′‐CTCCATAAAGCAGCCAAAGC‐3′), and carbohydrate‐responsive element‐binding protein (CHREBP) (Fwd: 5′‐CTTCAGCAGTGGGATCCTG‐3′; Rev: 5′‐ATCCAAGGGTCCAGAGCA‐3′). Messenger RNA expression results were normalized to *Hprt* expression and presented as a ratio relative to SED. HPRT was used as the reference gene because it is stable in the context of exercise (Cappelli et al. 2008).

### Mitochondrial DNA copy number

Total DNA was isolated from powdered gastrocnemius samples using the QIAamp DNA Mini Kit (Qiagen). Independent reactions were performed for mitochondrial DNA cytochrome c oxidase subunit I (COI) and nuclear 18S rDNA using the primers as detailed by Tal et al. (2009) with the LightCycler Carousel‐Based System (Roche). Primers used to amplify mouse DNA are as follows: 18S rDNA forward, 5′‐TAGAGGGACAAGTGGCGTTC‐3′; 18S rDNA reverse, 5′‐CGCTGAAGCCAGTCAGTGT‐3′; COI forward, 5′‐GCCCCAGTATAGCATTCCC‐3′; and COI reverse, 5′‐GTTCATCCTGTTCCTGCTCC‐3′. In brief, 5 ng of total DNA was used with the SYBR green I reaction system (Roche) according to the manufacturer's instructions. Temperature cycling program as detailed by Brown and Clayton (2002) with some modifications are as follows: initial denaturation at 95°C for 30 sec followed by 40 cycles at 95°C for 3 sec, 55°C for 6 sec then 72°C for 10 sec each cycle with ramping rate set at 20°C sec^−1^. Data were acquired at the end of the extensions at 72°C. At the end of the program, the homogeneity of the amplicons was confirmed by a melting curve analysis. Relative copy number are expressed as the ratio of mtDNA COI over 18S rDNA then rationalized to SED.

### Immunoblotting

Approximately 10 mg gastrocnemius samples were homogenized in 500 *μ*L cold RIPA lysis buffer with EDTA supplemented with protease and phosphatase inhibitor cocktail tablets (Roche). Insoluble matter was precipitated by centrifugation at 10,000 *g*, 4°C for 20 min and the lysate collected and protein content measured using the Coomassie Brilliant Blue solution (Nacalai Tesque, Kyoto, Japan). Lysate was adjusted with lysis buffer to achieve similar concentrations among samples followed by addition of 4× Laemmili buffer containing *β*‐mercaptoethanol. Samples (33 *μ*g protein) were subjected to electrophoresis followed by transfer to a nitrocellulose membrane. Membranes were incubated in Ponceau S stain then rinsed with excess water to remove unbound stain. These were then sandwiched in glass plates then photographed and digitized (LAS‐3000, Fujifilm, Tokyo, Japan). After digitization, membranes were washed in TBS‐T buffer to remove bound stain then followed by blocking in 2% BSA in TBS‐T. Membranes were cut at previously identified locations to separate PGC‐1*α* and *α*‐Tubulin containing regions. Respective primary antibody incubation was done with anti‐*α*‐Tubulin antibody and anti‐PGC‐1*α* antibody (Santa Cruz Biotechnology) overnight. Membranes were sufficiently washed and incubated with respective secondary antibodies linked to horseradish peroxidase (Dako, Glostrup, Denmark). Immunodetection by chemiluminescence (Western Lightning Plus ECL, PerkinElmer, Waltham, MA) was conducted according to the manufacturer's directions followed by visualization using the LAS‐3000 equipment. Quantification of total signals from Ponceau S, and protein‐specific chemiluminescent signals were performed using the coupled software (MultiGauge V.3.2, Fujifilm, Tokyo, Japan). Relative protein expression was obtained by comparing protein‐specific signals to total Ponceau S signal.

### Citrate synthase activity

Muscle lysates were diluted with the lysis buffer to achieve a similar protein concentration of 2 *μ*g *μ*L^−1^. The principle of the assay is based on the paper by Srere (1969). In a 96‐well plate, 4 *μ*L of adjusted lysate (8 *μ*g protein) was mixed with acetyl‐CoA (Roche, Indianapolis, IN) dissolved in 0.1 mol L^−1^ Tris‐HCl buffer at pH 8.0 and DTNB (Sigma‐Aldrich, Hamburg, Germany) dissolved in ethanol. After thorough mixing, deacetylase activity was measured at 412 nm on a kinetic program of a 96‐well plate reader for 2.5 min with 50‐sec interval between each reading. Citrate synthase reaction was initiated by the addition of oxaloacetate (Sigma‐Aldrich) dissolved in the same buffer. Final concentrations of reactants were as follows: 0.3 mmol L^−1^ acetyl‐CoA, 0.1 mmol L^−1^ DTNB, and 0.1 mmol L^−1^ oxaloacetate. The plate was immediately shaken and absorbance at 412 nm was read on a kinetic program with the duration of 10 min and an interval of 50 sec between each reading. Beyond this time, absorbance readings deviated from linearity. The change in absorbance before the addition of oxaloacetate was subtracted from the change in absorbance after the addition of oxaloacetate. Citrate synthase activity is expressed relative to protein loaded.

### Statistical analysis

Statistical analysis was performed using Prism 5.0 (Graphpad Software, La Jolla, CA). Time course data values of indirect calorimetry are presented as means. Average and cumulative values in different experiments are presented as mean ± SEM. One‐way analysis of variance (ANOVA) followed by Newman–Keuls post hoc test was applied to determine the presence of significant differences among groups. Student's *t*‐test was applied to within‐group pre‐ and postexercise data. Significance level was set at *α *= 0.05 and *P* values lower than *α* were considered significant.

## Results

### Body weight and sedentary‐state indirect calorimetry

**Figure 2:**
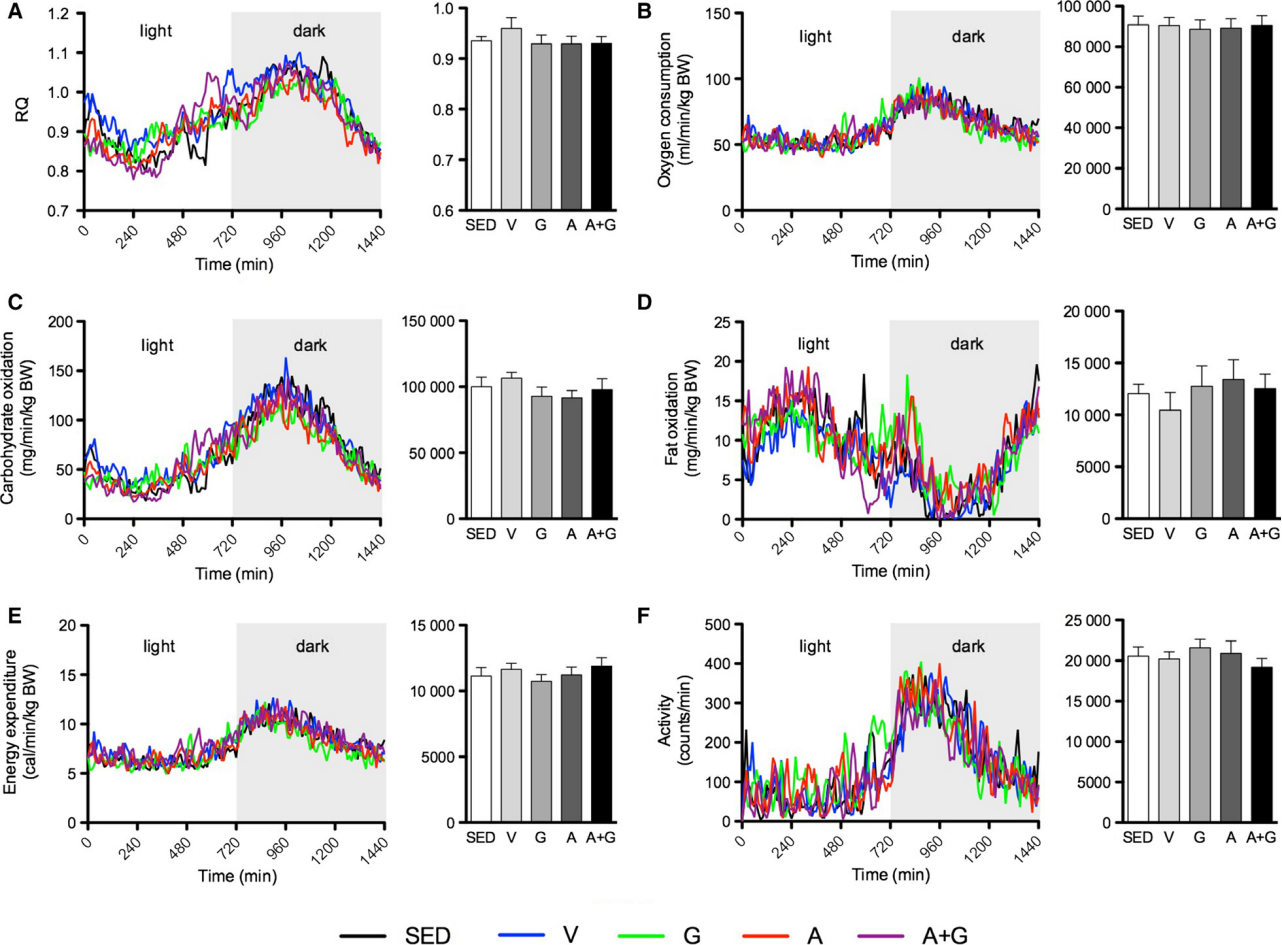
Combined pharmacological AMPK and PPARδ activation does not influence basal metabolism. Indirect calorimetry with activity measurement was conducted in the sedentary state. (A) Respiratory quotient (RQ), (B) oxygen consumption, (C) carbohydrate oxidation, (D) fat oxidation, (E) energy expenditure, and (F) spontaneous motor activity. The graph on the left depicts values at each time point while that on the right shows the average (only for RQ) or cumulative values within 24 h. Data are expressed as means ± SEM (*n* = 8–9). No significant differences were observed as analyzed by one‐way ANOVA.

The effects of combined pharmacological activation on body weight, organ weight, RQ, oxygen consumption, carbohydrate and fat oxidation, energy expenditure, and spontaneous motor activity were measured. No significant effect on body weight, gastrocnemius, and epididymal fat weights were observed (Table 1). However, liver weight was significantly increased in G (*P *<* *0.05) relative to SED, and in A+G (*P *<* *0.001) relative to all groups. No significant differences were observed in RQ, total oxygen consumption, carbohydrate and fat oxidation, energy expenditure, and spontaneous motor activity among groups (Fig. 2A–F).

**Table 1:** Body and organ weights

	SED	V	G	A	A+G
Body weight (g)	24.73 ± 0.360	25.36 ± 0.527	24.10 ± 0.471	24.80 ± 0.276	25.70 ± 0.435
Gastrocnemius (% of total BW)	0.5227 ± 0.014	0.5469 ± 0.013	0.5507 ± 0.013	0.5613 ± 0.014	0.5594 ± 0.006
Liver (% of total BW)	4.314 ± 0.07054^a^	4.554 ± 0.1232^a^	4.917 ± 0.1648^b^	4.530 ± 0.09468^a^	5.463 ± 0.08814^c^
Epididymal fat (% of total BW)	0.7654 ± 0.572	0.7063 ± 0.042	0.7054 ± 0.054	0.7886 ± 0.062	0.7541 ± 0.049

Different superscript notations indicate significant difference (*P *<* *0.05–0.001).

### Running endurance

**Figure 3:**
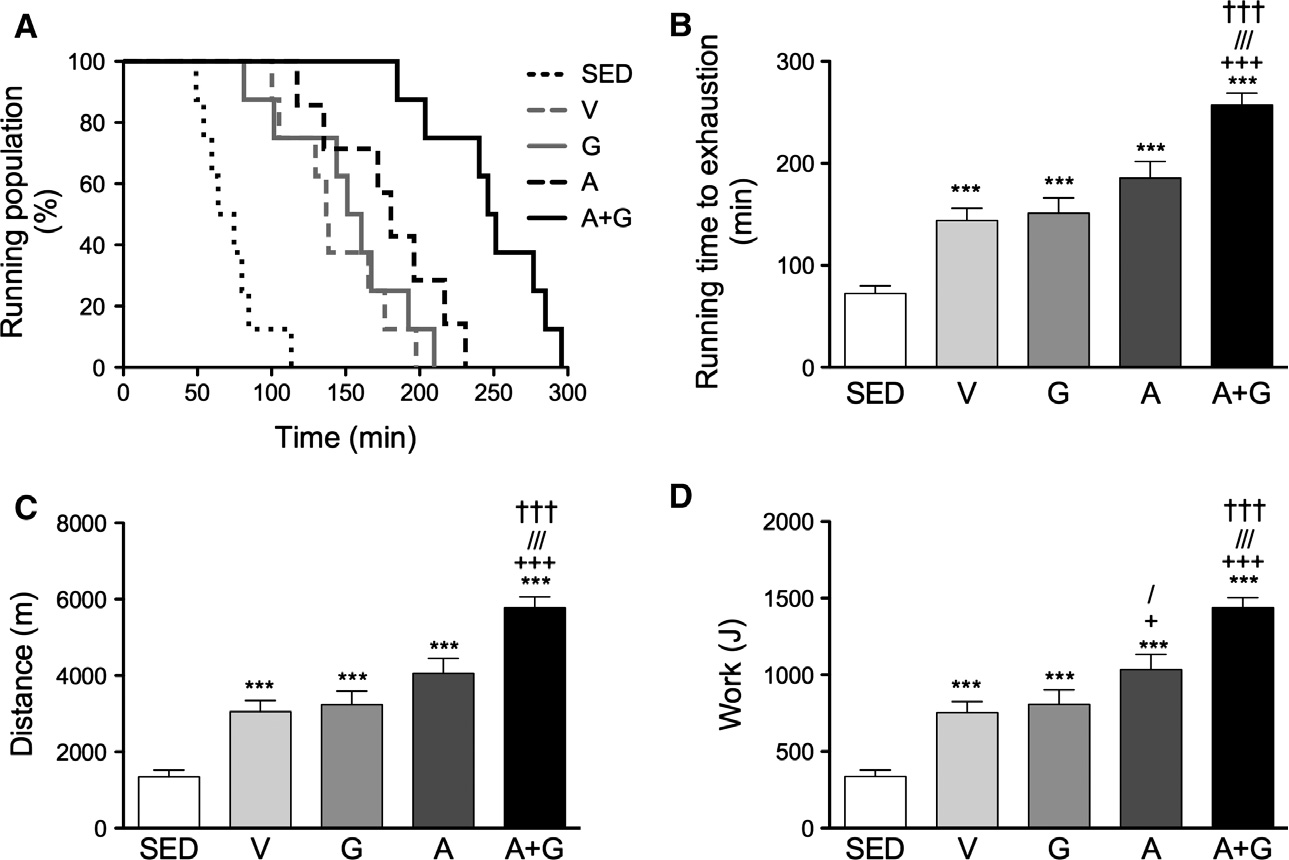
Combined pharmacological AMPK and PPARδ activation improves endurance in trained mice. Mice were subjected to exercise‐to‐exhaustion test. (A) Percentage of running population on the treadmill, (B) total running time to exhaustion, (C) total distance covered, and (D) total work done. Data are expressed as means ± SEM (*n* = 7–8). Significant differences were analyzed by one‐way ANOVA followed by Newman‐Keuls multiple comparison test. Asterisk (*), plus sign (+), slash (/) and dagger (†) represent significant difference relative to SED, V, G and A, respectively. Single symbols indicate *P* < 0.05 while triple symbols indicate *P* < 0.001.

To determine the effect of the activators on endurance in trained mice, mice were subjected to an exercise‐to‐exhaustion test. As expected, mice in the SED group were not able to run at the same time as the exercise‐trained groups (Fig. 3A and B). All the trained groups were able to run more than twice (*P *<* *0.001) the time to exhaustion of SED. Among them, only A+G was able to run longer (*P *<* *0.001) than V. A tendency for Group A to run longer was observed. The total distance covered until exhaustion was also calculated from the running time (Fig. 3C). However, work may be a more appropriate measure as it takes into account body weight. Indeed, calculating for work showed that A did significantly more (*P *<* *0.05) than both V and G however, A+G was still significantly higher (*P *<* *0.001) even to A (Fig. 3D). These results show that AICAR could potentiate endurance in trained mice. Furthermore, the combination of AICAR and GW0742 in A+G could further potentiate endurance than that imparted solely by AICAR.

### Exercise‐to‐exhaustion indirect calorimetry

**Figure 4:**
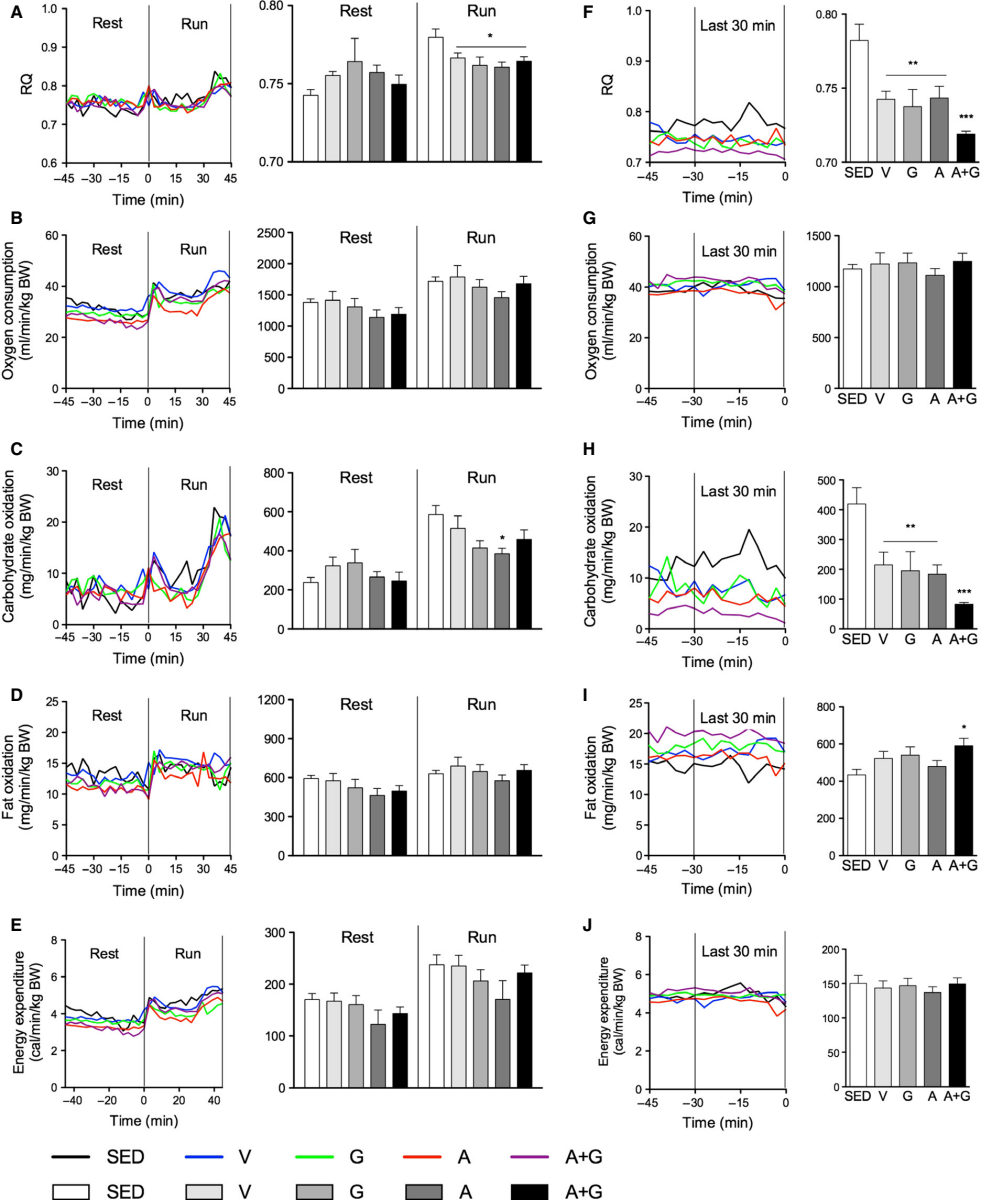
Combined pharmacological AMPK and PPARδ activation promotes enhanced substrate shift during prolonged exercise in trained mice. Indirect calorimetry was conducted before and during the exercise‐to‐exhaustion test. (A) Respiratory quotient (RQ), (B) oxygen consumption, (C) carbohydrate oxidation, (D) fat oxidation, and (E) energy expenditure. The same parameters were measured during the last 30 min until exhaustion: (F) RQ, (G) oxygen consumption, (H) carbohydrate oxidation, (I) fat oxidation, and (J) energy expenditure. Time course data are expressed as means while average RQ and cumulative values are expressed as means ± SEM (*n* = 6). Significant differences were analyzed by one‐way ANOVA followed by Newman‐Keuls multiple comparison test. *P < 0.05; **P < 0.01; and ***P < 0.001 relative to SED.

The effects of combined pharmacological activation on whole‐body metabolism were examined by indirect calorimetry before and during the running test. Only values obtained during the first 45 min of the run wherein SED was capable of completing were calculated to facilitate statistical comparison of all groups. No considerable differences were observed at each time point in the time course curves (Fig. 4A–E; curve) similar to the observation in sedentary‐state indirect calorimetry. Calculating for the average RQ, and total oxygen consumption and carbohydrate and fat oxidation before running, no significant differences were observed among the groups regardless of training status (Fig. 4A–E; rest bar). However, during the run, the exercise‐trained groups had significantly lower (*P *<* *0.05) average RQ compared to SED (Fig. 4A; run bar). Total oxygen consumption, fat oxidation, and energy expenditure did not show any significant differences (Fig. 4B, D and E; run bar). Only A had significantly decreased carbohydrate oxidation (*P *<* *0.05) relative to SED although G and A+G had a tendency to have lower total carbohydrate oxidation (Fig. 4C; run bar).

To visualize the changes in substrate oxidation before reaching exhaustion, the same calculation as above was applied to the last 10 time points corresponding to the last 30 min before exhaustion of each mice (Fig. 4F–J; curve). The average RQ was further decreased in all exercise‐trained groups with the highest decrease in A+G (Fig. 4F, bar). Total oxygen consumption was not different among groups (Fig. 4G; bar). Interestingly, total carbohydrate oxidation of all exercise‐trained groups showed a decrease (*P *<* *0.01–0.001) >50% that of SED (Fig. 4H; bar). Also, A+G had a further drop about 50% that of V, G, or A. Fat oxidation, on the other hand, was elevated (*P *<* *0.05) in A+G relative to SED in contrast to the lack of difference during the 45 min run (Fig. 4I; bar). This suggests an enhanced shift in fuel source during prolonged exercise especially near exhaustion as energy expenditure among all groups was not significantly different (Fig. 4J; bar).

### Serum glucose

**Figure 5:**
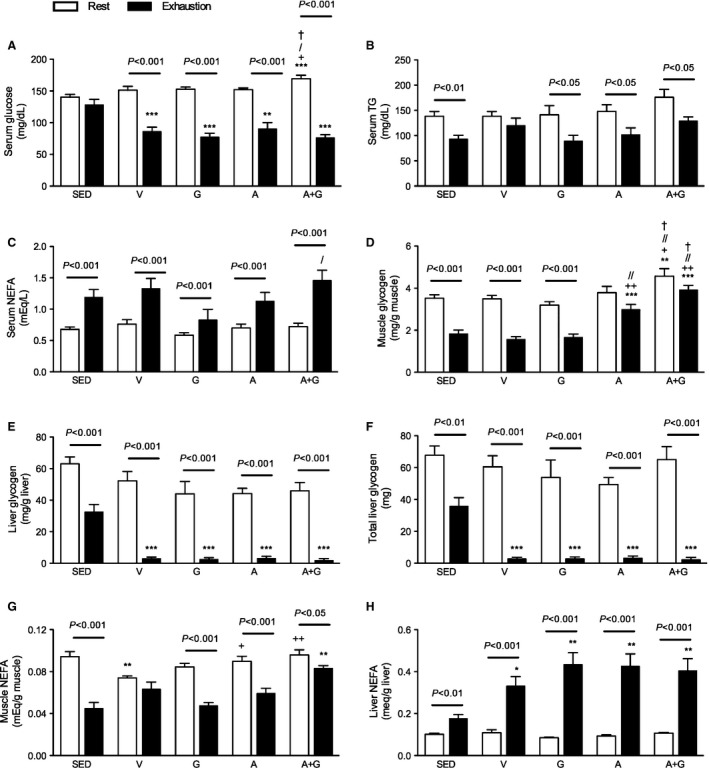
Combined pharmacological AMPK and PPARδ activation increases available substrates and muscle glycogen sparing in trained mice. Blood, gastrocnemius and liver were collected at rest and at the point of exhaustion. (A) Serum glucose, (B) serum TG, (C) serum NEFA, (D) muscle glycogen, (E) liver glycogen, (F) total liver glycogen, (G) muscle NEFA and (H) liver NEFA were measured. Data are expressed as means ± SEM (*n* = 8–9, at rest; 7–8, at exhaustion). Inter‐group significant differences were analyzed by one‐way ANOVA followed by Newman‐Keuls multiple comparison test while intra‐group differences were analyzed by unpaired Student's *t*‐test. Asterisk (*), plus sign (+), slash (/) and dagger (†) represent significant difference relative to SED, V, G and A, respectively. Single symbols, *P* < 0.05; double symbols, *P* < 0.05 while triple symbols, *P* < 0.001.

Serum glucose before exercise and after exhaustion was measured because hypoglycemia during exercise is implicated as one of the factors that lead to exercise cessation (Williams et al. 2013). Serum glucose before exercise was significantly increased in A+G relative to all the other groups. At the point of exhaustion, serum glucose was decreased (*P *<* *0.01–0.001) at about similar levels in all exercise‐trained groups irrespective of treatment compared to SED (Fig. 5A). And this decrease was significant (*P *<* *0.001) relative to the before‐exercise glucose levels. The SED group on the other hand did not have a significant decrease even at the point exhaustion.

### Serum TG and NEFA

Serum TG and NEFA are sources of fatty acids as energy source during exercise. Serum TG before exercise was similar among groups with A+G having a nonsignificant increase (Fig. 5B). No intergroup differences were observed at the point of exhaustion. Comparison of pre and postexercise values showed a significant decrease (*P *<* *0.05–0.01) in all groups except V.

Pre‐exercise serum NEFA was similar among the groups (Fig. 5C). At the point of exhaustion, serum NEFA significantly increased (*P *<* *0.001) relative to before exercise despite an apparent lower concentration in G. This low concentration was only significantly different (*P *<* *0.05) with A+G.

### Muscle and liver glycogen

Both muscle and hepatic glycogen were measured because glycogen plays a role in the maintenance of glucose homeostasis in the blood (Baldwin et al. 1973). Before exercise, the basal level of muscle glycogen was significantly elevated (*P *<* *0.05–0.01) in A+G relative to all groups. At the point of exhaustion, muscle glycogen in A and A+G remained elevated (*P *<* *0.01–0.001) compared to the other groups (Fig. 5D). Furthermore, A+G was significantly higher (*P *<* *0.05) than A. Comparison of pre and postexercise muscle glycogen showed that all groups had decreased. However, the decrease in A and A+G was not statistically significant compared with the other groups (*P *<* *0.001).

In the liver, pre‐exercise hepatic glycogen had a tendency to be lower in exercise‐trained groups (Fig. 5E). Because of increased liver weight in G and A+G (Table 1), calculating for absolute liver glycogen showed that SED and A+G had almost similar total glycogen while the other groups had lower content (Fig. 5F). At the point of exhaustion, all exercise‐trained groups had similar depleted glycogen stores about 15× less (*P *<* *0.001) than SED (Fig. 5E). All groups had decreased glycogen postexercise (*P *<* *0.001), but the decrease in SED did not drop to the same extent as in the exercise‐trained group.

### Muscle and liver NEFA

Because NEFA, albeit measured in plasma, delay the depletion of glycogen during exercise (Rennie et al. 1976), NEFA within the tissues were measured as no significant differences were observed in serum NEFA at least relative to SED at the point of exhaustion. Pre‐exercise muscle NEFA were similar in all groups except in V where a significant decrease (*P *<* *0.05–0.01) relative to SED, A and A+G was observed (Fig. 5G). However, at the point of exhaustion, muscle NEFA was increased (*P *<* *0.01) in A+G relative to SED. Moreover, A+G had 25% more NEFA in the gastrocnemius than other exercise‐trained groups. Muscle NEFA decreased after exercise however, in V where pre‐exercise NEFA was lower, the observed decrease was not statistically significant.

Liver NEFA, on the other hand, is the source of circulating NEFA and ketone bodies. Pre‐exercise values were similar among groups, however, at exhaustion, exercise‐trained groups significantly increased (*P *<* *0.001) relative to SED (Fig. 5H). An increase (*P *<* *0.01–0.001) in all groups relative to pre‐exercise values were observed but the level in SED was only about 50% that of the exercise‐trained groups.

### Gene expression in the muscle

**Figure 6:**
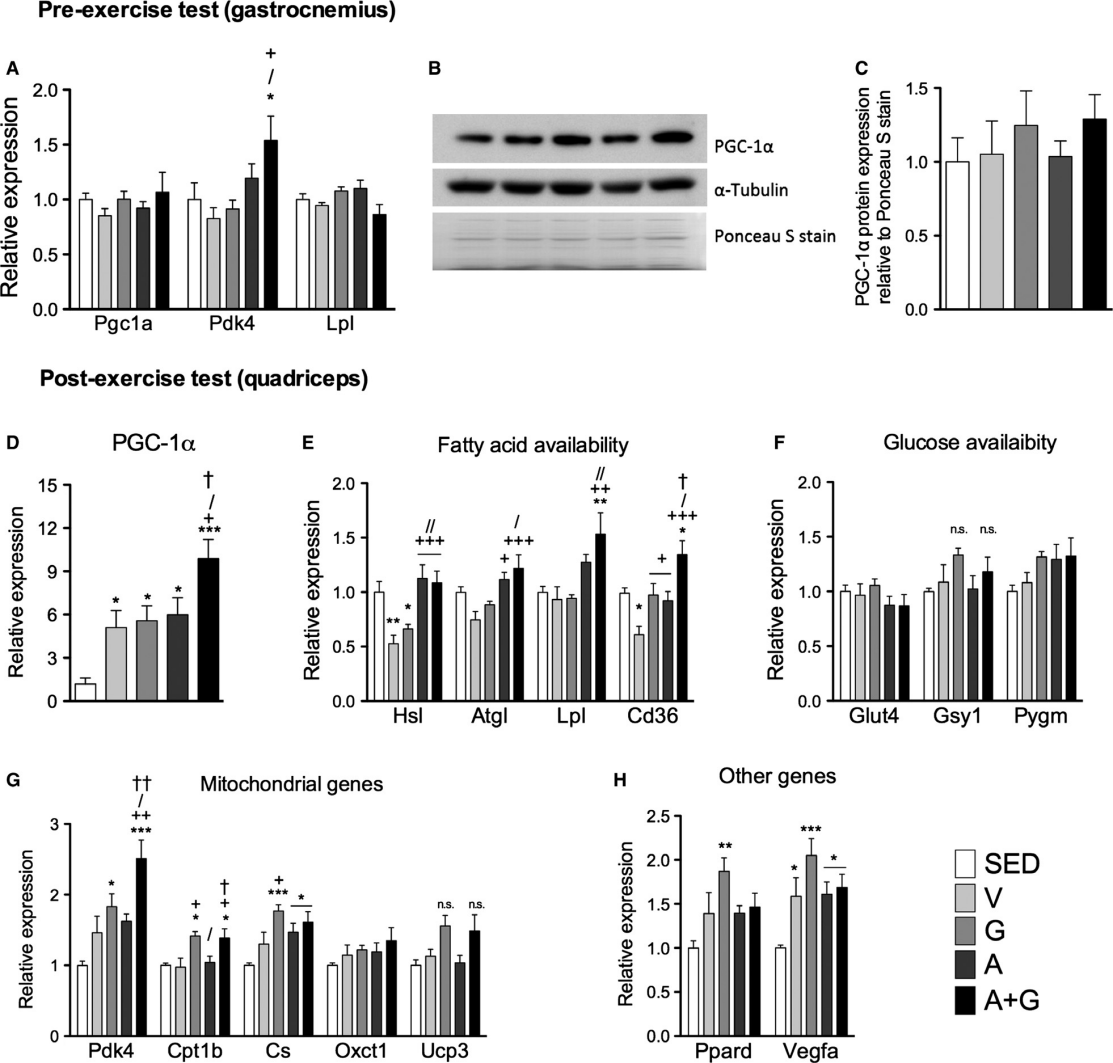
Combined pharmacological AMPK and PPARδ activation influences gene expression of substrate utilization related genes in skeletal muscle in trained mice. Pre‐exercise test (A) Pgc1a, Pdk4 and Lpl expression and, (B) representative immunoblot image of PGC‐1α and α‐Tubulin, and Ponceau S digitized image, and (C) relative PGC‐1α protein expression in gastrocnemius muscle. Post‐exercise test messenger RNA expression of (D) PGC‐1α and genes related to (E) fatty acid availability, (F) glucose availability, (G) mitochondrial oxidative metabolism, and (H) Ppard and Vegfa were measured. For mRNA expression, data were normalized to Hprt expression while for protein expression, data were normalized to total Ponceau S signal. Data were rationalized to SED, and expressed as means ± SEM (pre‐exercise test *n* = 6, postexercise test *n* = 7–9). Significant differences were analyzed by one‐way ANOVA followed by Newman‐Keuls multiple comparison test. Asterisk (*), plus sign (+), slash (/) and dagger (†) represent significant difference relative to SED, V, G and A, respectively. Single symbols, *P* < 0.05; double symbols, *P* < 0.01; while triple symbols, *P* < 0.001.

The transcriptional coactivator PGC‐1*α* has been associated with phenotypic changes leading to improved exercise. The effects of combined pharmacological activation on the gene expression of this coactivator were determined. It should be noted that the primer used in the study amplify the full‐length, well‐characterized transcript variant 1. Before the exercise test in the gastrocnemius, PGC‐1*α* mRNA expression was not different among groups regardless of training status (Fig. 6A). In A+G, *Pdk4* but not *Lpl* had significant upregulation (*P *<* *0.05). This elevation was not significant relative to A. Protein expression, on the other hand, tended to remain elevated after 72 h in the sedentary state in G and A+G, but not in other exercise groups (Fig. 6B and C). Comparison was made against total Ponceau S signal instead of commonly used loading controls. *α*‐Tubulin expression seemed to be influenced by exercise as its signal relative to total Ponceau S stain tended to be lower in exercise‐trained groups relative to SED and was further lowered by AICAR treatment (not shown). Likewise, *β*‐actin expression was variable despite similar amount protein loaded among samples as quantified (not shown).

After the exercise test in the quadriceps, exercise training significantly increased (*P *<* *0.05–0.001) PGC‐1*α* mRNA expression relative to SED (Fig. 6D). The lack of significant differences among V, G, and A suggests that individual drug administration does not increase *Pgc1a* expression above that of exercise training at least at the time point measured. Interestingly, combined pharmacological activation in A+G further increased (*P *<* *0.05) the *Pgc1a* levels than V, G, and A. Expression of mRNA of genes involved in fatty acid and glucose availability, mitochondrial genes, as well as PPAR*δ* and the angiogenic factor VEGFa were measured. Genes related to fatty acid availability were all highly expressed in A+G (Fig. 6E). In particular, AICAR treatment in A and A+G rescued the decrease of *Hsl* and *Atgl* compared to V. *Lpl* increased with AICAR treatment and A+G had the most robust upregulation (*P *<* *0.01). *Cd36* was decreased in V, but agonist treatment in G and A rescued it. Furthermore, it was further increased in A+G relative to G and A (*P *<* *0.05). Glucose availability‐related genes (*Glut4*,* Gsy1*, and *Pygm*) did not change. However, a tendency to increase with GW0742 treatment in *Gsy1* and with either agonist treatment in *Pygm* could be observed (Fig. 6F).

Messenger RNA expression of several mitochondrial genes was also measured at exhaustion (Fig. 6G). *Pdk4* was elevated by exercise and GW0742 in G induced a significant increase (*P *<* *0.05). The combination of the activators in A+G significantly elevated *Pdk4* (*P *<* *0.05–0.001) relative to all groups and almost 150% more than SED. *Cpt1b* was equally increased (*P *<* *0.05) in G and A+G, indicating no apparent significant effect of AMPK activation. *Cs* was increased (*P *<* *0.05–0.001) only in the drug treated groups although a tendency for V to increase could be observed. *Oxct1* did not increase with exercise or drug treatment. *Ucp3* increased in G and A+G but did not attain significance. The pattern of PGC‐1*α* protein expression in the exercise‐trained groups in particular was clearly reflected in the above mentioned genes. Exercise training increased the mRNA expression of PPAR*δ* (Fig. 6H). However, only G had the highest expression (*P *<* *0.01) relative to SED. Messenger RNA expression of VEGFA, another PGC‐1*α* target, was increased (*P *<* *0.05–0.001) by exercise training with G having the highest expression.

### Gene expression in the liver

**Figure 7:**
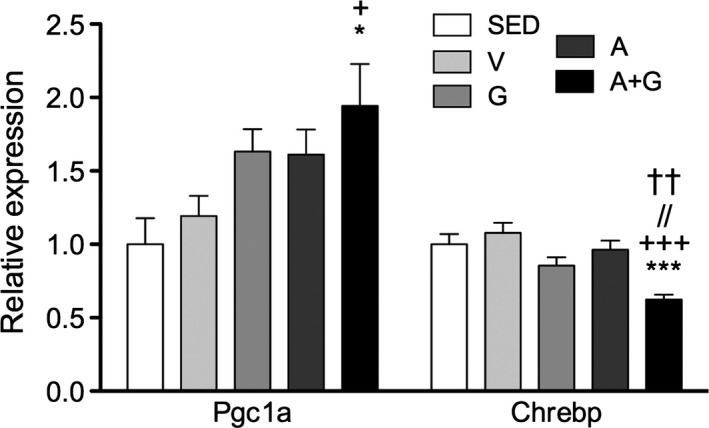
Combined pharmacological AMPK and PPARδ activation modifies hepatic mRNA expression of PGC‐1α and CHREBP in trained mice. After exhaustion, liver was analyzed for Pgc1a, and Chrebp expression. Data were normalized to Hprt expression, rationalized to SED, and expressed as means ± SEM (*n* = 7–8). Significant differences were analyzed by one‐way ANOVA followed by Newman‐Keuls multiple comparison test. Asterisk (*), plus sign (+), slash (/) and dagger (†) represent significant difference relative to SED, V, G and A, respectively. Single symbols, *P* < 0.05 while triple symbols, *P* < 0.001.

The maintenance of euglycemia during exercise is an important function of the liver. Transcription‐related factors known to influence the synthesis and storage of glucose in this organ were measured. At exhaustion, *Pgc1a* increased in all drug‐treated groups however, only A+G was significantly different (*P *<* *0.05) relative to both SED and V (Fig. 7). The lipogenic transcription factor CHREBP mRNA was significantly decreased (*P *<* *0.01–0.001) only in A+G. This was not observed even in the other exercise‐trained or drug‐treated groups. Both of these suggest increased synthesis and storage of glycogen with repression of glycolytic and lipogenic pathways in A+G (Burgess et al. 2006; Iizuka and Horikawa 2008).

### Mitochondrial density

**Figure 8:**
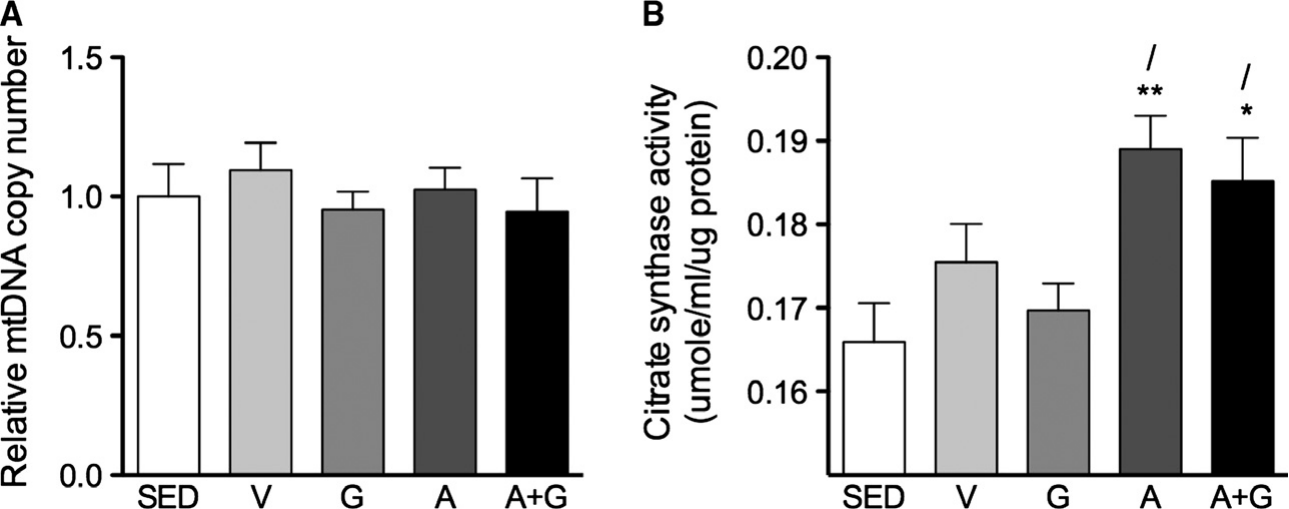
Pharmacological activation of AMPK but not PPARδ improved skeletal muscle mitochondrial density in trained mice. (A) Relative mtDNA copy number was measured by comparing the copy number of mitochondrial cytochrome oxidase I (COI) DNA with the copy number of nuclear 18S rDNA. (B) Citrate synthase activity was measured from muscle lysates. Mitochondrial DNA copy number data were rationalized to SED. Data are expressed as means ± SEM (*n* = 8–9). Significant differences were analyzed by one‐way ANOVA followed by Newman‐Keuls multiple comparison test. Asterisk (*), and slash (/) represent significant difference relative to SED, and G, respectively. Single symbols, *P* < 0.05 while double symbols, *P* < 0.01.

PGC‐1*α* is important in the stimulation of mitochondrial biogenesis (Austin and St‐Pierre 2012). Despite elevated gastrocnemius PGC‐1*α* protein expression in G and A+G (Fig. 6B and C), mtDNA copy number was not increased relative to SED (Fig. 8A). However, several studies emphasized that citrate synthase may be a better marker of mitochondrial biogenesis rather than mtDNA copy number (Kim et al. 2008; Larsen et al. 2012). Indeed, a tendency to have increased citrate synthase activity could be observed in all exercise‐trained groups (Fig. 8B). A moderate but nonsignificant increase was observed in V and G which was shadowed by the robust increase in citrate synthase activity (*P *<* *0.05–0.01) in AICAR‐treated groups.

## Discussion

This study demonstrated that combined pharmacological activation of AMPK and PPAR*δ* potentiates endurance in exercise‐trained mice. Although a small but significant potentiation was observed with AMPK activation, in contrast to Narkar et al. (2008), no potentiation was observed with PPAR*δ* activation. AMPK is a cellular sensor of energy status, while PPAR*δ* is a transcription factor known to induce a change toward an oxidative phenotype in metabolic tissues (Barish et al. 2006; Liu et al. 2011; Mihaylova and Shaw 2011). The lack of potentiation by PPAR*δ* agonism in two independent trials in contrast with Narkar et al. (2008) could be attributed to the differences in agonists, mice strain, and training and exhaustion test protocol. Despite this, our data are in agreement with Bueno Júnior et al. (2012) that combined activation of AMPK and PPAR*δ* with exercise training elicits phenotypic changes related to physical performance probably arise from the physical interaction of AMPK and PPAR*δ* (Narkar et al. 2008; Gan et al. 2011).

Whole‐body metabolism, both during sedentary and early phase of exercise, were not different among groups. However, in A+G, shift in fuel utilization from carbohydrate to fat was observed toward exhaustion without a change in energy expenditure. Shift in whole‐body fuel utilization has been attributed to PDK4, a PGC‐1*α* target gene, and mitochondrial protein responsible for the preferential *β*‐oxidation of fatty acids with a decline in glycolysis (Wende et al. 2005, 2007; Calvo et al. 2008). Indeed, despite an absence of changes in PGC‐1*α* mRNA transcription and an increase, albeit not significant, protein expression in the gastrocnemius of A+G before the exercise test, a small but significant elevation in *Pdk4* could observed. Furthermore, at exhaustion, *Pdk4* expression was increased significantly compared to all groups supporting the shift in fuel utilization toward exhaustion in A+G. In addition, PDK4 and PGC‐1*α* mRNA expression at exhaustion resembles that of fat oxidation toward exhaustion.

Combined pharmacological activation of AMPK and PPAR*δ* with exercise training increases substrate availability. Serum glucose, muscle glycogen, and hepatic glycogen were increased in A+G. With increased total available glucose in the form of free glucose, skeletal muscle and hepatic glycogen, the onset of hypoglycemia during exercise could be delayed. Increased basal glycogen in the muscle is associated with increased preference to fatty acid oxidation (Wong et al. 2014), while glycogen sparing during exercise both in muscle and liver is influenced by increased NEFA and substrate shift to fat by PDK4 thereby contributing to improved endurance (Rennie et al. 1976; Hickson et al. 1977; Pilegaard and Neufer 2004). AICAR has been shown to increase muscle glycogen synthesis (Holmes et al. 1999); however, at least in our experimental design, our data suggest that increased PDK4 through synergism with PPAR*δ* may be necessary to cause significant glycogen accumulation which explains the lack of increase in basal glycogen in A relative to A+G. Increased intramuscular NEFA at exhaustion in A+G is associated with elevated mRNA transcription of genes related to fatty acid uptake in this group as observed with significant elevation in *Hsl*,* Atgl*,* Lpl*, and *Cd36* permitting increased lipolysis from circulating triglycerides as well as uptake of fatty acids in the muscle. Taken together, the increase in *Pdk4* at basal and during exercise, transcription of fatty acid availability‐related genes, and consequently increased fatty acid availability during exercise would possibly delay the depletion of glucose and rapid onset of hypoglycemia consequently delaying fatigue and cessation of physical activity (Williams et al. 2013).

The liver not only stores energy in the form of glycogen, but also maintains energy homeostasis by maintaining normal blood glucose concentration through glycogenolysis and gluconeogenesis as well as ketone body synthesis during periods of energy deficit (Radziuk and Pye 2001; Mitra and Metcalf 2009; Cotter et al. 2013). The nonsignificant increase in total hepatic glycogen in A+G relative to other exercise‐trained groups may be influenced by the elevated expression of *Pgc1a* and *Chrebp*. Increased hepatic PGC‐1*α* leads to an improvement in oxidative metabolism, gluconeogenesis, and glycogenesis (Burgess et al. 2006; Finck and Kelly 2006), while CHREBP downregulation or silencing leads to decreased glycolysis and lipogenesis resulting in increased glycogen synthesis (Iizuka and Horikawa 2008). Moreover, elevated hepatic NEFA may suggest increased ketone body production as well as supply circulating fatty acids as observed in elevated serum NEFA, both contributing not only to muscle energy production, but also to sparing of circulating glucose and glycogen.

Mice in the exercise‐trained groups attained hypoglycemia at the point of exhaustion with concomitant exhaustion of liver glycogen not observed in SED. In SED, however, the absence of hypoglycemia despite reaching exhaustion suggests that other factors other than hypoglycemia prevented these mice from running longer (Piña et al. 2003), such as complete hepatic glycogen utilization, as well as other factors possibly developed or enhanced with training. Also, our observations indicate that muscle glycogen is dispensable in agreement with Pederson et al. (2005) using MGSKO mouse, a mouse model lacking muscle glycogen, that it is dispensable for exercise performance and concluded that muscle glycogen could limit exercise capacity only if the level of hepatic glycogen is low coinciding with our observations and Baldwin et al. (1973).

Exercise induces metabolic changes through the upregulation of transcription factors. As a major exercise‐inducible transcription factor coactivator, PGC‐1*α* has been shown to improve endurance through mitochondrial biogenesis, gluconeogenesis, triglyceride metabolism, glucose homeostasis, and muscle capillarization (Ryan and Hoogenraad 2007; Arany et al. 2008; Calvo et al. 2008; Lira et al. 2010; Narkar et al. 2011). In skeletal muscle, PPAR*δ* together with retinoid X receptor (RXR) activation controls its transcription while AMPK phosphorylates the PGC‐1*α* protein causing its self‐induced upregulation demonstrating the complex direct and indirect interaction of these two proteins (Schuler et al. 2006; Hondares et al. 2007; Jäger et al. 2007; Narkar et al. 2008; Gan et al. 2011). Contrary to expectation, *Pgc1a* was not elevated in the pre‐exercise gastrocnemius at the time of sampling. However, this is not surprising as reversion to basal levels toward 24 h postexercise in skeletal muscle following elevation immediately after exercise has been reported (Mathai et al. 2008). Because *Pgc1a* is induced by exercise, the length of time to exhaustion could influence the transcription levels in each group, with A+G running longer, thus having the highest expression level. This is also reflected by the mRNA expression of some of its target genes such as PDK4, LPL, and CD36 which are especially highest in A+G. *Oxct1*, a key enzyme in ketone body catabolism that is induced by exercise (Askew et al. 1975), tended to be highest in A+G. Furthermore, together with increased gene expression not exclusive to A+G (*Hsl*,* Atgl*,* Cpt1b*,* Cs*,* Vegfa*, and *Ppard*) would support the observed elevated intramuscular NEFA level at the point of exhaustion and the shift to whole‐body substrate oxidation to fat toward exhaustion in this group. Collectively, these findings suggest that combined pharmacological activation of AMPK and PPAR*δ* with exercise training results in a unique gene signature similar to that observed in oxidative muscle capable of improved delivery, uptake, and oxidation of fat and its preferential utilization. On one hand, because pre‐exercise test values likely reverted to baseline as seen in the lack of elevation in *Pgc1a* and *Lpl* and significant but modest elevation in *Pdk4* in A+G, protein expression rather than mRNA expression may reveal the participation of these genes in the observed endurance potentiation. On the other hand, postexercise data presented here may suggest that transcription induction of these genes during running had an additive or synergistic effect on endurance especially in A+G.

Exercise training through PGC‐1*α* promotes mitochondrial biogenesis consequently influencing oxidative metabolism and energy production (Austin and St‐Pierre 2012). The lack of mtDNA increase was not indicative of increased mitochondrial biogenesis as in muscle, CS activity is a more reliable marker (Larsen et al. 2012). Indeed, exercise‐trained groups had higher CS activity but interestingly, AICAR‐treated groups were significantly increased. Like PGC‐1*α* mRNA expression, CS activity presented may already be toward decline beyond 24 h similar to that observed by Leek et al. (2001). The observed small but significant elevation in work in A may be explained by the increase in CS activity. That of A+G, however, can be attributed to synergism of substrate availability, utilization shift, and increased mitochondrial density. Although not measured, metabolism of lactate and ketone bodies in A and A+G may coincide with increased mitochondrial density (Baldwin et al. 1978; Cotter et al. 2013).

## Conclusion

While pharmacological activation of AMPK potentiates endurance in trained and untrained mice as shown here and many studies (Murase et al. 2005, 2006; Narkar et al. 2008), combined activation with PPAR*δ* greatly potentiates endurance through the orchestration of transcriptional programs in the muscle and liver, leading to increased substrate availability, substrate shift to fat, and mitochondrial density.

## Conflict of Interest

None declared.

## References

[phy212625-bib-0001] Ahmadian, M. , J. M. Suh , N. Hah , C. Liddle , A. R. Atkins , M. Downes , et al. 2013 PPAR*γ* signaling and metabolism: the good, the bad and the future. Nat. Med. 99:557–566. doi: 10.1038/nm.3159. 23652116 10.1038/nm.3159 PMC3870016

[phy212625-bib-0002] Arany, Z. , S.‐Y. Foo , Y. Ma , J. L. Ruas , A. Bommi‐Reddy , G. Girnun , et al. 2008 HIF‐independent regulation of VEGF and angiogenesis by the transcriptional coactivator PGC‐1alpha. Nature 451:1008–1012. doi: 10.1038/nature06613. 18288196 10.1038/nature06613

[phy212625-bib-0003] Askew, E. W. , G. L. Dohm , and R. L. Huston . 1975 Fatty acid and ketone body metabolism in the rat: response to diet and exercise. J. Nutr. 105:1422–1432. 475 10.1093/jn/105.11.1422

[phy212625-bib-0004] Austin, S. , and J. St‐Pierre . 2012 PGC1*α* and mitochondrial metabolism–emerging concepts and relevance in ageing and neurodegenerative disorders. J. Cell Sci. 125:4963–4971. doi: 10.1242/jcs.113662. 23277535 10.1242/jcs.113662

[phy212625-bib-0005] Baldwin, K. M. , J. S. Reitman , R. L. Terjung , W. W. Winder , and J. O. Holloszy . 1973 Substrate depletion in different types of muscle and in liver during prolonged running. Am. J. Physiol. 225:1045–1050. 4745201 10.1152/ajplegacy.1973.225.5.1045

[phy212625-bib-0006] Baldwin, K. M. , A. M. Hooker , and R. E. Herrick . 1978 Lactate oxidative capacity in different types of muscle. Biochem. Biophys. Res. Commun. 83:151–157. 697805 10.1016/0006-291x(78)90410-2

[phy212625-bib-0007] Barish, G. D. , V. A. Narkar , and R. M. Evans . 2006 PPAR delta: a dagger in the heart of the metabolic syndrome. J. Clin. Invest. 116:590–597. doi: 10.1172/JCI27955. 16511591 10.1172/JCI27955 PMC1386117

[phy212625-bib-0008] Brown, T. A. , and D. A. Clayton . 2002 Release of replication termination controls mitochondrial DNA copy number after depletion with 2′,3′‐dideoxycytidine. Nucleic Acids Res. 30:2004–2010. 11972339 10.1093/nar/30.9.2004 PMC113833

[phy212625-bib-0009] Bueno Júnior, C. R. , L. C. Pantaleão , V. A. Voltarelli , L. H. M. Bozi , P. C. Brum , and M. Zatz . 2012 Combined effect of AMPK/PPAR agonists and exercise training in mdx mice functional performance. PLoS ONE 7:e45699. doi: 10.1371/journal.pone.0045699. 23029189 10.1371/journal.pone.0045699 PMC3448675

[phy212625-bib-0010] Burgess, S. C. , T. C. Leone , A. R. Wende , M. A. Croce , Z. Chen , A. D. Sherry , et al. 2006 Diminished hepatic gluconeogenesis via defects in tricarboxylic acid cycle flux in peroxisome proliferator‐activated receptor gamma coactivator‐1alpha (PGC‐1alpha)‐deficient mice. J. Biol. Chem. 281:19000–19008. doi: 10.1074/jbc.M600050200. 16670093 10.1074/jbc.M600050200 PMC3047410

[phy212625-bib-0011] Calvo, J. A. , T. G. Daniels , X. Wang , A. Paul , J. Lin , B. M. Spiegelman , et al. 2008 Muscle‐specific expression of PPAR*γ* coactivator‐1*α* improves exercise performance and increases peak oxygen uptake. J. Appl. Physiol. 104:1304–1312. doi: 10.1152/japplphysiol.01231.2007. 18239076 10.1152/japplphysiol.01231.2007

[phy212625-bib-0012] Cappelli, K. , M. Felicetti , S. Capomaccio , G. Spinsanti , M. Silvestrelli , and A. V. Supplizi . 2008 Exercise induced stress in horses: selection of the most stable reference genes for quantitative RT‐PCR normalization. BMC Mol. Biol. 9:49. doi: 10.1186/1471‐2199‐9‐49. 18489742 10.1186/1471-2199-9-49 PMC2412902

[phy212625-bib-0013] Chen, B. , Y. Ma , R. Meng , Z. Xiong , H. Wang , J. Zeng , et al. 2010 Activation of AMPK inhibits cardiomyocyte hypertrophy by modulating of the FOXO1/MuRF1 signaling pathway in vitro. Acta Pharmacol. Sin. 31:798–804. doi: 10.1038/aps.2010.73. 20581852 10.1038/aps.2010.73 PMC4007721

[phy212625-bib-0014] Cotter, D. G. , R. C. Schugar , and P. A. Crawford . 2013 Ketone body metabolism and cardiovascular disease. Am. J. Physiol. Heart Circ. Physiol. 304:H1060–H1076. doi: 10.1152/ajpheart.00646.2012. 23396451 10.1152/ajpheart.00646.2012 PMC3625904

[phy212625-bib-0015] Desvergne, B. , and W. Wahli . 1999 Peroxisome proliferator‐activated receptors: nuclear control of metabolism. Endocr. Rev. 20:649–688. doi: 10.1210/edrv.20.5.0380. 10529898 10.1210/edrv.20.5.0380

[phy212625-bib-0016] Finck, B. N. , and D. P. Kelly . 2006 PGC‐1 coactivators: inducible regulators of energy metabolism in health and disease. J. Clin. Invest. 116:615–622. doi: 10.1172/JCI27794. 16511594 10.1172/JCI27794 PMC1386111

[phy212625-bib-0017] Frayn, K. N. 1983 Calculation of substrate oxidation rates in vivo from gaseous exchange. J. Appl. Physiol. 55:628–634. 6618956 10.1152/jappl.1983.55.2.628

[phy212625-bib-0018] Gan, Z. , E. M. Burkart‐Hartman , D.‐H. Han , B. Finck , T. C. Leone , E. Y. Smith , et al. 2011 The nuclear receptor PPAR*β*/*δ* programs muscle glucose metabolism in cooperation with AMPK and MEF2. Genes Dev. 25:2619–2630. doi: 10.1101/gad.178434.111. 22135324 10.1101/gad.178434.111 PMC3248683

[phy212625-bib-0019] Hawley, S. A. , D. A. Pan , K. J. Mustard , L. Ross , J. Bain , A. M. Edelman , et al. 2005 Calmodulin‐dependent protein kinase kinase‐beta is an alternative upstream kinase for AMP‐activated protein kinase. Cell Metab. 2:9–19. doi: 10.1016/j.cmet.2005.05.009. 16054095 10.1016/j.cmet.2005.05.009

[phy212625-bib-0020] Hickson, R. C. , M. J. Rennie , R. K. Conlee , W. W. Winder , and J. O. Holloszy . 1977 Effects of increased plasma fatty acids on glycogen utilization and endurance. J Appl Physiol Respir Environ Exerc Physiol 43:829–833. 591476 10.1152/jappl.1977.43.5.829

[phy212625-bib-0021] Holmes, B. F. , E. J. Kurth‐Kraczek , and W. W. Winder . 1999 Chronic activation of 5′‐AMP‐activated protein kinase increases GLUT‐4, hexokinase, and glycogen in muscle. J. Appl. Physiol. 87:1990–1995. 10562646 10.1152/jappl.1999.87.5.1990

[phy212625-bib-0022] Hondares, E. , I. Pineda‐Torra , R. Iglesias , B. Staels , F. Villarroya , and M. Giralt . 2007 PPAR*δ*, but not PPAR*α*, activates PGC‐1*α* gene transcription in muscle. Biochem. Biophys. Res. Commun. 354:1021–1027. doi: 10.1016/j.bbrc.2007.01.092. 17275789 10.1016/j.bbrc.2007.01.092

[phy212625-bib-0023] Horman, S. , G. Browne , U. Krause , J. Patel , D. Vertommen , L. Bertrand , et al. 2002 Activation of AMP‐activated protein kinase leads to the phosphorylation of elongation factor 2 and an inhibition of protein synthesis. Curr. Biol. 12:1419–1423. 12194824 10.1016/s0960-9822(02)01077-1

[phy212625-bib-0024] Iizuka, K. , and Y. Horikawa . 2008 ChREBP: a glucose‐activated transcription factor involved in the development of metabolic syndrome. Endocr. J. 55:617–624. 18490833 10.1507/endocrj.k07e-110

[phy212625-bib-0025] Jäger, S. , C. Handschin , J. St.‐Pierre , and B. M. Spiegelman . 2007 AMP‐activated protein kinase (AMPK) action in skeletal muscle via direct phosphorylation of PGC‐1*α* . PNAS 104:12017–12022. doi: 10.1073/pnas.0705070104. 17609368 10.1073/pnas.0705070104 PMC1924552

[phy212625-bib-0026] Jensen, T. E. , A. J. Rose , S. B. Jørgensen , N. Brandt , P. Schjerling , J. F. P. Wojtaszewski , et al. 2007 Possible CaMKK‐dependent regulation of AMPK phosphorylation and glucose uptake at the onset of mild tetanic skeletal muscle contraction. Am. J. Physiol. Endocrinol. Metab. 292:E1308–E1317. doi: 10.1152/ajpendo.00456.2006. 17213473 10.1152/ajpendo.00456.2006

[phy212625-bib-0027] Kim, M. J. , C. Jardel , C. Barthélémy , V. Jan , J. P. Bastard , S. Fillaut‐Chapin , et al. 2008 Mitochondrial DNA content, an inaccurate biomarker of mitochondrial alteration in human immunodeficiency virus‐related lipodystrophy. Antimicrob. Agents Chemother. 52:1670–1676. doi: 10.1128/AAC.01449‐07. 18332166 10.1128/AAC.01449-07 PMC2346614

[phy212625-bib-0028] Krämer, D. K. , L. Al‐Khalili , B. Guigas , Y. Leng , P. M. Garcia‐Roves , and A. Krook . 2007 Role of AMP kinase and PPARdelta in the regulation of lipid and glucose metabolism in human skeletal muscle. J. Biol. Chem. 282:19313–19320. doi: 10.1074/jbc.M702329200. 17500064 10.1074/jbc.M702329200

[phy212625-bib-0029] Kurth‐Kraczek, E. J. , M. F. Hirshman , L. J. Goodyear , and W. W. Winder . 1999 5′ AMP‐activated protein kinase activation causes GLUT4 translocation in skeletal muscle. Diabetes 48:1667–1671. 10426389 10.2337/diabetes.48.8.1667

[phy212625-bib-0030] Larsen, S. , J. Nielsen , C. N. Hansen , L. B. Nielsen , F. Wibrand , N. Stride , et al. 2012 Biomarkers of mitochondrial content in skeletal muscle of healthy young human subjects. J. Physiol. (Lond.) 590:3349–3360. doi: 10.1113/jphysiol.2012.230185. 22586215 10.1113/jphysiol.2012.230185 PMC3459047

[phy212625-bib-0031] Leek, B. T. , S. R. Mudaliar , R. Henry , O. Mathieu‐Costello , and R. S. Richardson . 2001 Effect of acute exercise on citrate synthase activity in untrained and trained human skeletal muscle. Am. J. Physiol. Regul. Integr. Comp. Physiol. 280:R441–R447. 11208573 10.1152/ajpregu.2001.280.2.R441

[phy212625-bib-0032] Lefebvre, P. , G. Chinetti , J.‐C. Fruchart , and B. Staels . 2006 Sorting out the roles of PPAR alpha in energy metabolism and vascular homeostasis. J. Clin. Invest. 116:571–580. doi: 10.1172/JCI27989. 16511589 10.1172/JCI27989 PMC1386122

[phy212625-bib-0033] Li, Y. , S. Xu , M. M. Mihaylova , B. Zheng , X. Hou , B. Jiang , et al. 2011 AMPK phosphorylates and inhibits SREBP activity to attenuate hepatic steatosis and atherosclerosis in diet‐induced insulin‐resistant mice. Cell Metab. 13:376–388. doi: 10.1016/j.cmet.2011.03.009. 21459323 10.1016/j.cmet.2011.03.009 PMC3086578

[phy212625-bib-0034] Lira, V. A. , C. R. Benton , Z. Yan , and A. Bonen . 2010 PGC‐1alpha regulation by exercise training and its influences on muscle function and insulin sensitivity. Am. J. Physiol. Endocrinol. Metab. 299:E145–E161. doi: 10.1152/ajpendo.00755.2009. 20371735 10.1152/ajpendo.00755.2009 PMC2928513

[phy212625-bib-0035] Liu, S. , B. Hatano , M. Zhao , C.‐C. Yen , K. Kang , S. M. Reilly , et al. 2011 Role of peroxisome proliferator‐activated receptor delta}/{beta in hepatic metabolic regulation. J. Biol. Chem. 286:1237–1247. doi: 10.1074/jbc.M110.138115. 21059653 10.1074/jbc.M110.138115 PMC3020731

[phy212625-bib-0036] Lusk, G. 1924 Animal calorimetry Twenty‐Fourth Paper. analysis of the oxidation of mixtures of carbohydrate and fat. J. Biol. Chem. 59:41–42.

[phy212625-bib-0037] Mathai, A. S. , A. Bonen , C. R. Benton , D. L. Robinson , and T. E. Graham . 2008 Rapid exercise‐induced changes in PGC‐1alpha mRNA and protein in human skeletal muscle. J. Appl. Physiol. 105:1098–1105. doi: 10.1152/japplphysiol.00847.2007. 18653753 10.1152/japplphysiol.00847.2007

[phy212625-bib-0038] Maughan, R. J. 1999 Nutritional ergogenic aids and exercise performance. Nutr. Res. Rev. 12:255–280. doi: 10.1079/095442299108728956. 19087454 10.1079/095442299108728956

[phy212625-bib-0039] Mihaylova, M. M. , and R. J. Shaw . 2011 The AMPK signalling pathway coordinates cell growth, autophagy and metabolism. Nat. Cell Biol. 13:1016–1023. doi: 10.1038/ncb2329. 21892142 10.1038/ncb2329 PMC3249400

[phy212625-bib-0040] Mitra, V. , and J. Metcalf . 2009 Metabolic functions of the liver. Anaesth. Intensive Care Med. 10:334–335. doi: 10.1016/j.mpaic.2009.03.011.

[phy212625-bib-0041] Mungai, P. T. , G. B. Waypa , A. Jairaman , M. Prakriya , D. Dokic , M. K. Ball , et al. 2011 Hypoxia triggers AMPK activation through reactive oxygen species‐mediated activation of calcium release‐activated calcium channels. Mol. Cell. Biol. 31:3531–3545. doi: 10.1128/MCB.05124‐11. 21670147 10.1128/MCB.05124-11 PMC3165558

[phy212625-bib-0042] Murase, T. , S. Haramizu , A. Shimotoyodome , A. Nagasawa , and I. Tokimitsu . 2005 Green tea extract improves endurance capacity and increases muscle lipid oxidation in mice. Am. J. Physiol. Regul. Integr. Comp. Physiol. 288:R708–R715. doi: 10.1152/ajpregu.00693.2004. 15563575 10.1152/ajpregu.00693.2004

[phy212625-bib-0043] Murase, T. , S. Haramizu , A. Shimotoyodome , I. Tokimitsu , and T. Hase . 2006 Green tea extract improves running endurance in mice by stimulating lipid utilization during exercise. Am. J. Physiol. Regul. Integr. Comp. Physiol. 290:R1550–R1556. doi: 10.1152/ajpregu.00752.2005. 16410398 10.1152/ajpregu.00752.2005

[phy212625-bib-0044] Narkar, V. A. , M. Downes , R. T. Yu , E. Embler , Y.‐X. Wang , E. Banayo , et al. 2008 AMPK and PPARdelta agonists are exercise mimetics. Cell 134:405–415. doi: 10.1016/j.cell.2008.06.051. 18674809 10.1016/j.cell.2008.06.051 PMC2706130

[phy212625-bib-0045] Narkar, V. A. , W. Fan , M. Downes , R. T. Yu , J. W. Jonker , W. A. Alaynick , et al. 2011 Exercise and PGC‐1*α*‐independent synchronization of type I muscle metabolism and vasculature by ERR*γ* . Cell Metab. 13:283–293. doi: 10.1016/j.cmet.2011.01.019. 21356518 10.1016/j.cmet.2011.01.019 PMC3084588

[phy212625-bib-0046] Pederson, B. A. , C. R. Cope , J. M. Schroeder , M. W. Smith , J. M. Irimia , B. L. Thurberg , et al. 2005 Exercise capacity of mice genetically lacking muscle glycogen synthase in mice, muscle glycogen is not essential for exercise. J. Biol. Chem. 280:17260–17265. doi: 10.1074/jbc.M410448200. 15711014 10.1074/jbc.M410448200

[phy212625-bib-0047] Pilegaard, H. , and P. D. Neufer . 2004 Transcriptional regulation of pyruvate dehydrogenase kinase 4 in skeletal muscle during and after exercise. Proc. Nutr. Soc. 63:221–226. 15294034 10.1079/pns2004345

[phy212625-bib-0048] Piña, I. L. , C. S. Apstein , G. J. Balady , R. Belardinelli , B. R. Chaitman , B. D. Duscha , et al. 2003 Exercise and heart failure a statement from the American Heart Association Committee on exercise, rehabilitation, and prevention. Circulation 107:1210–1225. doi: 10.1161/01.CIR.0000055013.92097.40. 12615804 10.1161/01.cir.0000055013.92097.40

[phy212625-bib-0049] Radziuk, J. , and S. Pye . 2001 Hepatic glucose uptake, gluconeogenesis and the regulation of glycogen synthesis. Diabetes Metab. Res. Rev. 17:250–272. 11544610 10.1002/dmrr.217

[phy212625-bib-0050] Reilly, S. M. , and C.‐H. Lee . 2008 PPAR delta as a therapeutic target in metabolic disease. FEBS Lett. 582:26–31. doi: 10.1016/j.febslet.2007.11.040. 18036566 10.1016/j.febslet.2007.11.040 PMC2275052

[phy212625-bib-0051] Rennie, M. J. , W. W. Winder , and J. O. Holloszy . 1976 A sparing effect of increased plasma fatty acids on muscle and liver glycogen content in the exercising rat. Biochem J 156:647–655. 949346 10.1042/bj1560647 PMC1163799

[phy212625-bib-0052] Ryan, M. T. , and N. J. Hoogenraad . 2007 Mitochondrial‐nuclear communications. Annu. Rev. Biochem. 76:701–722. doi: 10.1146/annurev.biochem.76.052305.091720. 17227225 10.1146/annurev.biochem.76.052305.091720

[phy212625-bib-0053] Schuler, M. , F. Ali , C. Chambon , D. Duteil , J.‐M. Bornert , A. Tardivel , et al. 2006 PGC1alpha expression is controlled in skeletal muscles by PPARbeta, whose ablation results in fiber‐type switching, obesity, and type 2 diabetes. Cell Metab. 4:407–414. doi: 10.1016/j.cmet.2006.10.003. 17084713 10.1016/j.cmet.2006.10.003

[phy212625-bib-0100] Shackelford, D. B. , and R. J. Shaw . 2009 The LKB1‐AMPK pathway: metabolism and growth control in tumour suppression. Nat. Rev. Cancer 9:563‐575. doi:10.1038/nrc2676. 19629071 10.1038/nrc2676 PMC2756045

[phy212625-bib-0054] Srere, P. A. 1969 [1] Citrate synthase: [EC 4.1.3.7. Citrate oxaloacetate‐lyase (CoA‐acetylating)] Pp. 3–11 in Lowenstein J. M., ed. Methods in enzymology, citric acid cycle. Academic Press, New York, USA.

[phy212625-bib-0055] Tal, M. C. , M. Sasai , H. K. Lee , B. Yordy , G. S. Shadel , and A. Iwasaki . 2009 Absence of autophagy results in reactive oxygen species‐dependent amplification of RLR signaling. PNAS 106:2770–2775. doi: 10.1073/pnas.0807694106. 19196953 10.1073/pnas.0807694106 PMC2650341

[phy212625-bib-0056] Wang, Y.‐X. , C.‐L. Zhang , R. T. Yu , H. K. Cho , M. C. Nelson , C. R. Bayuga‐Ocampo , et al. 2004 Regulation of muscle fiber type and running endurance by PPAR*δ* . PLoS Biol. 2:e294. doi: 10.1371/journal.pbio.0020294. 15328533 10.1371/journal.pbio.0020294 PMC509410

[phy212625-bib-0057] Wende, A. R. , J. M. Huss , P. J. Schaeffer , V. Giguère , and D. P. Kelly . 2005 PGC‐1*α* coactivates PDK4 gene expression via the orphan nuclear receptor ERR*α*: a mechanism for transcriptional control of muscle glucose metabolism. Mol. Cell. Biol. 25:10684–10694. doi: 10.1128/MCB.25.24.10684‐10694.2005. 16314495 10.1128/MCB.25.24.10684-10694.2005 PMC1316952

[phy212625-bib-0058] Wende, A. R. , P. J. Schaeffer , G. J. Parker , C. Zechner , D.‐H. Han , M. M. Chen , et al. 2007 A role for the transcriptional coactivator PGC‐1alpha in muscle refueling. J. Biol. Chem. 282:36642–36651. doi: 10.1074/jbc.M707006200. 17932032 10.1074/jbc.M707006200

[phy212625-bib-0059] Williams, J. H. , T. W. Batts , and S. Lees . 2013 Reduced muscle glycogen differentially affects exercise performance and muscle fatigue. ISRN Physiol. 2013:1–8. doi: 10.1155/2013/371235.

[phy212625-bib-0060] Winder, W. W. , and D. G. Hardie . 1996 Inactivation of acetyl‐CoA carboxylase and activation of AMP‐activated protein kinase in muscle during exercise. Am. J. Physiol. 270:E299–E304. 8779952 10.1152/ajpendo.1996.270.2.E299

[phy212625-bib-0061] Wong, K. E. , C. R. Mikus , D. H. Slentz , S. E. Seiler , K. L. DeBalsi , O. R. Ilkayeva , et al. 2014 Muscle‐specific overexpression of PGC‐1a does not augment metabolic improvements in response to exercise and caloric restriction. Diabetes 64:1532–1543. doi: 10.2337/db14‐0827 25422105 10.2337/db14-0827 PMC4407850

[phy212625-bib-0062] Yang, J. , L. Craddock , S. Hong , and Z.‐M. Liu . 2009 AMP‐activated protein kinase suppresses LXR‐dependent sterol regulatory element‐binding protein‐1c transcription in rat hepatoma McA‐RH7777 cells. J. Cell. Biochem. 106:414–426. doi: 10.1002/jcb.22024. 19125418 10.1002/jcb.22024

